# Relative supersaturation values distinguish between feline urinary and non-urinary foods and align with expected urine analytes contributions to uroliths

**DOI:** 10.3389/fvets.2023.1167840

**Published:** 2023-08-04

**Authors:** Elizabeth M. Morris, Allison P. McGrath, John Brejda, Dennis E. Jewell

**Affiliations:** ^1^Hill’s Pet Nutrition, Inc., Topeka, KS, United States; ^2^Alpha Statistical Consulting, Inc., Lincoln, NE, United States; ^3^Department of Grain Science and Industry, Kansas State University, Manhattan, KS, United States

**Keywords:** struvite, calcium oxalate, magnesium ammonium phosphate, feline, relative supersaturation, urolith

## Abstract

**Introduction:**

Uroliths are concretions formed in the urinary tract. These can be problematic in humans and companion animals such as cats. Magnesium ammonium phosphate (struvite) and calcium oxalate (CaOx) are the most common forms of uroliths. The relative supersaturation (RSS) is a relative risk index of crystal formation. Here, an updated program for calculating RSS, EQUIL-HL21, was used to detect differences in RSS values when cats were fed foods formulated for urinary and non-urinary conditions. In addition, the contributions of urinary analytes to RSS values were examined via regression analyses.

**Methods:**

Historical data from feeding trials including foods indicated for use in urinary or non-urinary conditions were analyzed for nutrient composition and urinary parameters. RSS was calculated by EQUIL-HL21. The relationship between RSS values calculated by EQUIL-HL21 and urinary analytes was examined by regression models, which were selected by R^2^ and stepwise methods.

**Results:**

Cats that consumed urinary foods had significantly greater levels of urinary sodium and chloride compared with those that consumed non-urinary foods, consistent with the greater amounts of sodium and chloride in the urinary foods. Those that consumed non-urinary foods had higher urine pH, ammonium, potassium, phosphorus, magnesium, oxalate, citrate, and sulfate. Struvite RSS value and number of urinary crystals were significantly lower in cats fed the urinary foods. Mean CaOx RSS values were similar in both foods, though the number of CaOx crystals were significantly higher in cats that consumed non-urinary foods. A model predicting the natural log of struvite RSS values indicated that these values would increase with increasing urine pH, ammonium, chloride, calcium, phosphorus, and magnesium, and would decrease with increasing urine citrate and sulfate. CaOx RSS was predicted to increase as urinary chloride, calcium, and oxalates increased, and would decrease as urine pH, sodium, phosphorus, citrate, and sulfate increased.

**Discussion:**

These analyses demonstrate that the EQUIL-HL21 program can accurately detect expected differences between foods formulated for urinary and non-urinary indications. Regression models showed the eight urinary analytes that, respectively, contribute to the predicted RSS values for struvite and CaOx.

## 1. Introduction

Uroliths are concretions formed in the urinary tract under conditions that are influenced by pH conditions and concentrations of various types of inorganic and organic materials (1). Formation of uroliths is a common condition in cats, accounting for 13 to 28% of cases of feline lower urinary tract diseases (2). The most common uroliths reported in cats are composed of calcium oxalate (CaOx) or magnesium ammonium phosphate (struvite) (3–6). Age, sex, and breed are predisposing factors in the formation of certain types of uroliths (6, 7). Cats with kidney stones have a significantly shorter lifespan than those without (12.5 vs. 15.2 years) (8), and the prevalence of chronic kidney disease (CKD) in cats is significantly higher in cats with urolithiasis than in those without, though it is not known whether urolithiasis is a predictor or consequence of CKD (9).

The relative proportion of CaOx and struvite uroliths in affected cats has changed over time, with a decrease in the proportion of struvite uroliths and increase in CaOx uroliths (2). For example, an analysis of feline uroliths showed that the respective proportions of CaOx and struvite uroliths were 2 and 78% in 1981 and 41 and 49% in 2007 (4). Similar trends and proportions were found in different geographical locations, including Minnesota (4, 10), California (6, 10), Canada (5, 11), and the Benelux countries (12). Examination of age, sex, breed, and reproductive status of cats over this time period indicate that those factors are not responsible for the observed changes in the prevalences of struvite and CaOx uroliths (13).

In general, limiting the amounts of urolith precursors in food, controlling for urine pH, and diluting the urine can help to prevent urolith formation (1). Struvite uroliths can be prevented by feeding an acidifying maintenance food with low magnesium and phosphorus (14). From the early 1980s to the early 2000s, the pet food industry shifted to low magnesium, urine-acidifying foods, which may have caused the decrease in the proportion of struvite uroliths and increase in the proportion of CaOx uroliths. It is reported that feeding foods that acidify the urine results in increased urinary calcium excretion (15, 16), which raises the risk of CaOx uroliths (17). However, results from a more recent study indicated that acidification of the urine pH in ranges representative of those resulting from consumption of most commercial foods did not increase the risk of CaOx crystallization, although foods resulting in lower urine pH did reduce the risk of struvite crystallization (18).

Acidification of urine appears to reduce struvite formation but may increase the risk of CaOx urolith formation (2, 4). In fact, existing struvite uroliths can be dissolved in about 2–5 weeks by consumption of foods that are low in magnesium and acidify the urine (14, 19–21). Magnesium can form a complex with oxalate, which inhibits the association of oxalate with calcium and thus decreases CaOx urolith formation (22). In a large study evaluating food and urolith composition, an increased risk of struvite uroliths in cats was observed with foods containing high fiber, calcium, phosphorus, magnesium, sodium, potassium, or chloride and that were formulated to increase urine pH, whereas cats had a higher risk of CaOx uroliths when fed foods with significantly lower protein, sodium, potassium, or moisture and that were formulated to reduce urine pH (23). While CaOx uroliths cannot be dissolved using a food-based intervention (2), there is some evidence that CaOx uroliths can be prevented with specially formulated food (24) that also increases the concentration of glycosaminoglycans in urine, which inhibit the growth and aggregation of CaOx crystals (25).

Relative supersaturation (RSS) provides a risk index of crystallization via a computer algorithm into which the concentrations of several urinary solutes and pH are entered (1). Several different methods have been developed to calculate urine supersaturation (26–30). EQUIL is a program originally coded in FORTRAN (31) that has been considered the gold standard for RSS calculations in humans (32). EQUIL was updated to the BASIC coding language in 1985, yielding EQUIL2 (33). Another update, EQUIL93, added more ions to be evaluated (34), but those are beyond the number analyzed in standard practice, thus limiting its use in clinical settings. The EQUIL-HL21 program is a Windows-based update of EQUIL2 that can be used on modern computers, uses new coefficients for urinary solutes, has recently been shown to be similar with EQUIL2, and is available in the cited publication (35).

The present study tested the hypothesis that EQUIL-HL21 could show differences in CaOx and struvite RSS values for cat foods that are or are not indicated for urinary conditions. It was expected that consumption of urinary foods would result in RSS for struvite and CaOx at the lower end of the metastable range (1–2.5 for struvite and < 12 for CaOx uroliths in cats (36)) and that non-urinary foods would result in significantly higher struvite and CaOx RSS values. In addition, the relationship between urine analytes and the RSS values for CaOx and struvite was examined in order to evaluate the biological relevance and relationship between the urine analytes and their contributions to these RSS values in the context of animal-to-animal variation.

## 2. Materials and methods

### 2.1. Study design

A search of historical data from food trials conducted at Hill’s Pet Nutrition, Inc. was completed to find those with available urinalysis data from July 30, 2010 to March 13, 2020. All trial protocols had been approved by the Hill’s Institutional Animal Care and Use Committee and complied with Hill’s Global Animal Welfare Policy and the United States National Research Council guidelines (37). Cats were housed individually at Hill’s Pet Nutrition Center in condos that opened into a bigger room for unrestricted daily socialization on non-collection days. Food was provided once daily with access for approximately 22 h each day and water access was unlimited. Trials were selected based on foods from multiple brands that were either (1) indicated for use in cats with urinary conditions, including those indicated to dissolve struvite stones (“urinary” foods), or (2) not indicated for use in cats with urinary conditions, including wellness foods from a diverse range of brands and therapeutic nutrition that was not specifically indicated for urinary conditions (“non-urinary” foods). Selected trials also included at least 10 cats fed over a period of at least 7 days (mean 20.7 days; range 7–28 days). Data for 130 observations from cats fed 10 urinary foods and 127 observations from cats fed 10 non-urinary foods were chosen for this analysis. One of the non-urinary foods was used in two trials. Some observations were from the same animals that were used in more than one trial, but because each trial or feeding period was independent of other trials, these data were included.

### 2.2. Sample collection and analysis

Samples of study foods were collected and analyzed prior to each trial. Nutrient compositions of the foods were determined by Eurofins (Lancaster, PA, United States) using standard AOAC methods (38).

On sample collection days, cats that were acclimated to urine collection and in their individual condos voluntarily voided urine into litter boxes with inert beads (Polypropylene Plastic Pellets Virgin PP only; SmartCat Box, Providence House Mfg. Inc., Lake Elsinore, CA, United States) over a 24-h period. [While the amount of collected urine was typically sufficient within 24 h, study protocols allowed the cats to remain in the study if they had not urinated within 24 h (up to 72 h). The sample was pulled when the collected urine volume was sufficient.] During collection periods, litter boxes were checked four times daily for feces, which was removed along with replacement of the beads to prevent contamination of urine. Litter boxes contained a drain hole and tubing to allow urine to directly collect in a thymol (Alfa Aesar, Thermo Fisher Scientific, Ward Hill, MA, United States)-treated container (to prevent bacterial growth) in a closed system (to prevent evaporation) that was housed within a 37°C water bath. Following the 24-h collection period, urine was moved to the laboratory and immediately analyzed for urine pH by potentiometric measurement using a pH meter (B30PCI, SympHony, VWR, Radnor, PA, United States) that was calibrated on each day of use. Urinalysis, including evaluation of urine sediment for crystals, was performed following pH analysis on the day of collection prior to freezing. Samples were then frozen at – 20^o^ C until individual analyses were conducted using validated methods within 14 days of collection. Ammonium (39) and oxalate (40) were analyzed as previously described. Minerals (including calcium, magnesium, phosphorus, potassium, and sodium) were analyzed by inductively coupled plasma spectrometry (41), and sulfate and chloride were analyzed by ion chromatography (42), both adapted from AOAC methods. Citrate was determined via an enzymatic colorimetric assay (Boehringer Mannheim/R-Biopharm, Washington, MO, United States). These data were then used to calculate RSS via EQUIL-HL21 (Hill’s Pet Nutrition, Inc., Topeka, KS) (35).

### 2.3. Statistical analysis

For analyses of urinary and non-urinary foods, summary statistics and linear model-based hypothesis tests of mean differences are presented. For the hypothesis test, two different linear models were used. If the variances were significantly different for the two categories of food, then a linear mixed model was used with each food category as a fixed effect and separate variances were estimated for each category. If the variances were similar for the two categories of food, then a linear mixed model was used with food category as a fixed-effect and animal and animal x food category as random effects. It was not possible to combine both models into one because the full model often did not converge. Effects were considered significant when *p* ≤ 0.05.

To examine the relationship between CaOx or struvite RSS and urine analytes, multiple regression models were developed using PROC REG in SAS^®^ version 9.4 (SAS Institute, Cary, NC, United States) that could predict RSS values for struvite and CaOx generated by EQUIL-HL21 software. An optimal model is one that, for a given number of predictor variables, produces the minimum error sum of squares, or, equivalently, the maximum *R*^2^ statistic (43). To achieve this, two selection methods were used. One method was RSQUARE, which calculates the *R*^2^ statistic of all one-variable models, then for all two-variable models, then three-variable models, etc. Because the *R*^2^ statistic generally increases as more variables are added to a model regardless of their statistical significance, other criteria are needed to identify the optimal model. To achieve this goal, Mallows’ Cp statistic was used. Mallows recommends the model where Cp first approaches p, which is the number of predictor variables plus the intercept. The parameter estimates are unbiased when the correct model is chosen, and this is reflected in Cp near *p*.

The other selection method employed to examine the relationship between RSS and urine analytes was a stepwise procedure in which variables are added one by one to the model. The *F* statistic for a variable to be added had to be significant at the SLENTRY = level. For this analysis, a variable had to be significant at the *α* = 0.10 probability level to be included in the model. Variables were added to the model until no new variables meet the SLENTRY criteria. Variables were retained in the final model if the significance level to stay in the model was also < 0.10.

The inclusion of many predictor variables in a regression model can often result in multicollinearity, a high degree of correlation among two or more predictor variables (43). The existence of multicollinearity can inflate the variances of predicted values as well as the variances of the slope estimates and can result in estimates with unstable coefficients with incorrect signs or magnitudes. To check for multicollinearity in the models, the variance inflation factor (VIF) was used. The VIF is defined as 1/(1 − *R*^2^*i*), where *R*^2^*i* is the coefficient of determination for the regression of the *i*th independent variable on all other independent variables. The VIF statistic shows how multicollinearity has increased the instability of the coefficient estimates. Although there is no formal criteria for determining the magnitude of the VIF statistic that indicates poorly estimated coefficients, some suggest that values exceeding 10 may be cause for concern (43).

The procedure used to select the best model (PROC REG) uses least-squares regression, which does not allow for inclusion of random effects such as animal-to-animal variation. Therefore, to determine if the model could be improved, and whether slopes estimates were affected by accounting for animal-to-animal variation, the final model was reanalyzed using PROC GLIMMIX in SAS. The PROC GLIMMIX model included the final set of predictor variables as continuous covariates and animal as a random effect. Two criteria were used to assess the results: comparing the residual error variance from the model with and without a random animal term in the model, and the COVTEST option in PROC GLIMMIX, which produces a likelihood ratio test to determine whether the added variance component is significantly different from 0.

## 3. Results

### 3.1. Characteristics of cats used in the studies

A total of 78 cats, in whom 257 observations were recorded, were utilized in the studies chosen for this analysis (Table 1).

**Table 1:** Characteristics of cats in the included studies.

Characteristic	Urinary foods (*n* = 10)	Non-urinary foods (*n* = 10)
Animals, *n*	43	35
Age, years	6.2 ± 2.4	6.0 ± 2.4
Sex, *n*		
NM	18	15
SF	25	20
Body weight, kg	4.9 ± 0.7	4.9 ± 0.8
Total observations, *n*	130	127
Feeding studies per animal, *n*	3.0 ± 1.9	3.6 ± 1.9
Feeding studies per animal, median (range)	1.9 (1–6)	1.9 (1–6)

Data are mean ± standard deviation unless otherwise indicated. One observation represents one collection from one particular cat.

NM, neutered male; SF, spayed female.

### 3.2. Comparisons of urinary and non-urinary foods

Comparison of the nutrient composition between urinary (*n* = 10 foods) and non-urinary (*n* = 10) foods showed that the urinary foods were significantly higher in energy, carbohydrate, potassium, sodium, and chloride, while non-urinary foods were higher in crude fat, calcium, phosphorus, and magnesium (Table 2). There was no significant difference between the two types of foods for crude protein, crude fiber, or sulfate. Nutrient compositions of the individual foods included in this analysis are in Supplementary Table 1.

**Table 2:** Least-squares means ± standard errors for nutrient composition on a dry matter basis of feline foods that are or are not indicated for urinary conditions.

Nutrient composition	Urinary foods (*n* = 10)	Non-urinary foods (*n* = 10)	*p*-value
Energy, kcal/kg^1^	4,744 ± 56	4,333 ± 57	< 0.001
Crude protein	36.7 ± 0.59	38.1 ± 0.63	0.132
Crude fat	17.9 ± 0.27	20.5 ± 0.69	< 0.001
Carbohydrate (NFE)	36.3 ± 1.00	31.6 ± 1.06	0.002
Crude fiber	2.2 ± 0.17	2.5 ± 0.18	0.165
Ash	7.1 ± 0.18	7.3 ± 0.19	0.568
Calcium	0.85 ± 0.04	1.29 ± 0.04	< 0.001
Phosphorus	0.80 ± 0.01	1.07 ± 0.04	< 0.001
Potassium	0.93 ± 0.01	0.90 ± 0.01	0.001
Magnesium	0.07 ± 0.01	0.11 ± 0.002	< 0.001
Sodium	0.90 ± 0.04	0.45 ± 0.02	< 0.001
Chloride	1.62 ± 0.06	0.77 ± 0.03	< 0.001
Sulfate	0.96 ± 0.08	0.74 ± 0.12	0.122

Units are % unless otherwise indicated.

1 In the event that energy content was not analyzed in a food, the published metabolizable energy value was used.

NFE, nitrogen-free extract.

Cats that were fed urinary foods (*n* = 130 observations) had significantly higher sodium and chloride in urine compared with cats fed non-urinary foods (*n* = 127 observations; Table 3), which is consistent with the higher levels of sodium and chloride present in the urinary foods. Feeding non-urinary foods resulted in significantly higher urine pH, ammonium, potassium, phosphorus, magnesium, oxalate, citrate, and sulfate. There was no significant difference in urine calcium between the two groups.

**Table 3:** Least-squares means ± standard errors for urine parameters measured in cats consuming urinary or non-urinary products.

Urine parameters	Urinary food (*n* = 130)	Non-urinary food (*n* = 127)	*p*-value
Calcium oxalate RSS	3.33 ± 0.32	3.71 ± 0.37	0.071
Calcium oxalate crystals, count^1^	1	11	0.003
Struvite RSS	1.04 ± 0.17	4.48 ± 0.42	<0.001
Struvite crystals, count	8	47	<0.001
pH	6.15 ± 0.05	6.72 ± 0.05	<0.001
Ammonium, mM	190 ± 7.0	220 ± 7.0	0.002
Sodium, mM	220 ± 6.0	110 ± 6.0	<0.001
Potassium, mM	106 ± 5.0	200 ± 6.0	<0.001
Chloride, mM	250 ± 11.0	150 ± 6.0	<0.001
Calcium, mM	1.1 ± 0.1	1.2 ± 0.1	0.093
Phosphorus, mM	50.0 ± 2.0	80.0 ± 2.0	<0.001
Magnesium, mM	3.2 ± 0.2	4.5 ± 0.2	<0.001
Oxalate, mM	0.8 ± 0.04	1.2 ± 0.04	<0.001
Citrate, mM	1.2 ± 0.4	4.3 ± 0.4	<0.001
Sulfate, mM	80 ± 3.5	90 ± 0.4	0.016

1 Actual count of the number of samples in which crystals were observed under microscopic observation.

RSS, relative supersaturation.

The struvite RSS of urine from cats fed non-urinary foods was more than 4-fold greater compared with those fed the urinary foods (*p* < 0.001), and the number of struvite crystals present in urine were about 6-fold higher (*p* < 0.001; Table 3). Although the group means for CaOx RSS were not significantly different for the two food categories (*p* = 0.071), the CaOx crystal count was 11-fold higher (*p* = 0.003) in cats that had consumed non-urinary foods.

### 3.3. Predictive models for struvite RSS values

Both the RSQUARE and stepwise methods selected the same models for predicting struvite RSS values, with an *R*^2^ of 0.58 (Table 4). The Cp statistic first decreased below the number of predictor variables with a five-predictor variable model. The slope estimates indicated that struvite RSS increased as urine pH, magnesium, and oxalates increased, and struvite RSS decreased as urine sodium and citrate increased (Table 5). All of the predictor variables in this model have a VIF value < 2, indicating that multicollinearity is not a problem with this model. There was no benefit in accounting for animal-to-animal variation with this model, as the animal variance component was not significantly different from 0 (*p* = 0.325), and the residual error variance remained largely unchanged with the inclusion of a random animal term.

**Table 4:** Urinary predictor variables selected and the associated *R*^2^ and Mallows’ Cp statistics for the RSQUARE selection method, and predictor urinary predictor variables selected by the stepwise selection method for predicting struvite relative supersaturation.

Number of variables	Selection method: RSQUARE	*R* ^2^	Mallows’ Cp	Selection method: stepwise
1	pH	0.470	57.3	pH
2	pH, magnesium	0.520	30.6	pH, magnesium
3	pH, magnesium, citrate	0.558	12.0	pH, magnesium, citrate
4	pH, magnesium, oxalate, citrate	0.570	7.1	pH, magnesium, oxalate, citrate
5	pH, sodium, magnesium, oxalate, citrate	0.578	4.8	pH, sodium, magnesium, oxalate, citrate

**Table 5:** Slope estimates, standard errors (SE), *p*-values, and variance inflation factor (VIF) for a five-predictor variable model selected for predicting struvite relative supersaturation.

Variable	Slope estimates	Standard errors (SE)	*p*-value	Variance inflation factor (VIF)
Intercept	−32.26	2.26	<0.001	0
pH	5.16	0.35	<0.001	1.49
Sodium	−3.93	1.70	0.022	1.02
Magnesium	519	103.3	<0.001	1.91
Oxalate	1,278	518.3	0.014	1.92
Citrate	−286	51.3	<0.001	1.59

For evaluating bias in the model, predicted versus observed struvite RSS values were plotted (Supplementary Figure 1). Struvite RSS values were relatively linear from 0 to 8, with the prediction equations underestimating the observed RSS beginning around 8. The curvature in a plot of the residuals versus observed values for struvite RSS indicate bias at both low and high values, with the model underestimating the predicted value at very low (< 0.5) RSS values and overestimating at very high (> 8) RSS values (Supplementary Figure 2).

In order to improve the predictive performance of the model, struvite RSS was natural log-transformed and the RSQUARE and stepwise selection methods were repeated. Both methods selected the same eight-predictor variable model with an *R*^2^ of 0.94 (Table 6). The slope estimates indicated that predicted struvite RSS values increased with increasing urine pH, ammonium, chloride, calcium, phosphorus, and magnesium, and that struvite RSS decreased with increasing urine citrate and sulfate (Table 7). Two variables, urinary ammonium and urinary sulfate, had VIF values > 3, which are well below the threshold of 10. Although these two analytes were strongly correlated with each other (*r* = 0.784), the magnitude of this correlation was not strong enough to appreciably affect the coefficient estimates in the model, and so multicollinearity is not considered to be a serious problem with this model.

**Table 6:** Urinary predictor variables selected and the associated *R*^2^ and Mallows’ Cp statistics for the RSQUARE selection method, and urinary predictor variables selected by the stepwise selection method for predicting the natural log of struvite relative supersaturation.

Number of Variables	Selection method: RSQUARE	*R* ^2^	Mallows’ Cp	Selection method: stepwise
1	pH	0.659	1,196	pH
2	pH, magnesium	0.845	414	pH, magnesium
3	pH, ammonium, magnesium	0.884	254	pH, ammonium, magnesium
4	pH, ammonium, magnesium, citrate	0.916	121	pH, ammonium, magnesium, citrate
5	pH, ammonium, phosphorus, magnesium, citrate	0.936	37	pH, ammonium, phosphorus, magnesium, citrate
6	pH, ammonium, chloride, phosphorus, magnesium, citrate	0.940	24	pH, ammonium, chloride, phosphorus, magnesium, citrate
7	pH, ammonium, chloride, phosphorus, magnesium, citrate, sulfate	0.943	12.2	pH, ammonium, chloride, phosphorus, magnesium, citrate, sulfate
8	pH, ammonium, chloride, calcium, phosphorus, magnesium, citrate, sulfate	0.944	8.6	pH, ammonium, chloride, calcium, phosphorus, magnesium, citrate, sulfate

**Table 7:** Slope estimates, standard error (SE), *p*-values, and variance inflation factor (VIF) for an eight-predictor variable model for predicting the natural log of struvite relative supersaturation.

Variable	Slope estimates	Standard errors (SE)	*p*-value	Variance inflation factor (VIF)
Intercept	−21.1	0.37	<0.001	0
pH	2.87	0.05	<0.001	1.35
Ammonium	6.26	0.61	<0.001	3.11
Chloride	0.97	0.27	<0.001	1.22
Calcium	69.1	29.1	0.018	1.20
Phosphorus	10.4	1.22	<0.001	1.29
Magnesium	301	15.2	<0.001	1.88
Citrate	−89.2	7.62	<0.001	1.54
Sulfate	−4.16	1.15	<0.001	3.04

A plot of the natural log of predicted struvite RSS values and natural log of observed struvite RSS values showed good agreement between the two (Supplementary Figure 3). Since struvite RSS values on a non-logarithmic scale are of more interest to clinicians, the antilog values for predicted and observed struvite RSS were plotted (Supplementary Figure 4). These values generally followed the 1:1 line, particularly at lower values, indicating that there was an absence of bias in the model. The residuals plot shows that they were mainly randomly distributed around 0 (Supplementary Figure 5).

### 3.4. Predictive model for CaOx RSS values

The RSQUARE and stepwise methods were also used to select the best model for predicting CaOx RSS using urine analytes with an *R*^2^ of 0.94 (Table 8). Mallows’ Cp statistic first decreased below the number of predictor variables with an eight-variable model. Both methods selected the same models, with the exception of the seven-predictor variable model, for which the RSQUARE method chose urinary phosphorus but the stepwise method chose urinary potassium. The slope estimates indicate that CaOx RSS increased as urine chloride, calcium, and oxalates increased, and that CaOx RSS decreased as urine pH, sodium, phosphorus, citrate, and sulfate increased (Table 9). All of the predictor variables in this model had a VIF value < 10 (43), indicating that multicollinearity is not a problem with this model. There was no benefit from accounting for animal-to-animal variation with this model because the animal variance component was not significantly different from 0 (*p* = 0.917), and the residual error variance remained largely unchanged with the inclusion of a random animal term.

**Table 8:** Predictor variables selected and the associated *R*^2^ and Mallows’ Cp statistics for the RSQUARE selection method, and predictor variables selected by the stepwise selection method for predicting calcium oxalate relative supersaturation.

Number of Variables	Selection method: RSQUARE	*R* ^2^	Mallows’ Cp	Selection method: stepwise
1	Calcium	0.762	642	Calcium
2	Calcium, citrate	0.799	511	Calcium, citrate
3	Calcium, oxalate, citrate	0.901	132	Calcium, oxalate, citrate
4	Potassium, calcium, oxalate, citrate	0.923	51.8	Potassium, calcium, oxalate, citrate
5	Sodium, potassium, calcium, oxalate, citrate	0.927	39.9	Sodium, potassium, calcium, oxalate, citrate
6	Sodium, potassium, chloride, calcium, oxalate, citrate	0.933	18.9	Sodium, potassium, chloride, calcium, oxalate, citrate
7	Sodium, chloride, calcium, potassium, oxalate, citrate, sulfate	0.936	11.7	Sodium, potassium, chloride, calcium, oxalate, citrate, sulfate
8	pH, sodium, chloride, calcium, potassium, oxalate, citrate, sulfate	0.937	7.9	pH, sodium, chloride, calcium, potassium, oxalate, citrate, sulfate

**Table 9:** Slope estimates, standard error (SE), *p*-values, and variance inflation factor (VIF) for an eight-predictor variable model selected for predicting calcium oxalate relative supersaturation.

Variable	Slope estimates	Standard errors (SE)	*p*-Value	Variance inflation factor (VIF)
Intercept	1.31	0.80	0.101	0
pH	−0.23	0.12	0.049	1.84
Sodium	−5.59	1.09	<0.001	4.59
Chloride	3.41	1.13	0.003	5.17
Calcium	2,970	59.2	<0.001	1.18
Phosphorus	−11.4	2.47	<0.001	1.27
Oxalate	2,820	178	<0.001	2.48
Citrate	−243	15.5	<0.001	1.57
Sulfate	−10.6	1.64	<0.001	1.45

Bias in the eight-predictor variable model for predicting CaOx RSS was evaluated. The predicted and observed values mainly fell along a 1:1 diagonal line, with only a few predicted values below the line, indicating that the bias does not appear to be very strong (Supplementary Figure 6). Similarly, the residuals were generally randomly scattered along the x-axis, although above the value of 10 most of the residuals were positive, indicating that predicted values were slightly underestimated in this range (Supplementary Figure 7).

## 4. Discussion

This study used the new EQUIL-HL21 program to compare CaOx and struvite RSS values and urine parameters in cats following the consumption of urinary or non-urinary foods. These data establish that EQUIL-HL21 can detect differences in urinary and non-urinary foods where expected. The ideal RSS is ≤ 1 for dissolution (undersaturation) of struvite uroliths in cats (36). For prevention, the metastable range has been reported as 1–2.5 for struvite (36), with a mean of ≤ 1.8 as the midpoint of the metastable saturation zone. The urinary foods tested here showed a struvite RSS of 1.04, while the non-urinary foods had a struvite RSS of 4.49. These results are consistent with the RSS values for dissolution and prevention as well as with the commercial claims that the urinary foods can dissolve struvite uroliths. For CaOx uroliths in cats, the ideal RSS for prevention is < 12 (metastable) (36), with a mean of ≤ 6 as the midpoint of the metastable saturation zone. Both the urinary and non-urinary foods assessed in this study had CaOx RSS values < 4 and so are in the metastable range. The CaOx RSS values for the urinary and non-urinary foods in this study did not significantly differ despite an 11-fold difference in CaOx crystal formation. A possible explanation for this is that foods designed to prevent CaOx uroliths may include inhibitors that are not captured in the RSS calculation. RSS includes a limited number of inputs, and thus does not capture all known inhibitors to crystallization often used in formulations. For example, some urine proteins, such as glycosaminoglycans, osteopontin, nephrocalcin, and prothrombin fragment-1, can inhibit CaOx urolith formation (44, 45). Future work should examine the contributions of these factors to RSS values.

Significantly higher levels of sodium and chloride in urine were observed when cats were fed urinary foods compared with non-urinary foods, consistent with the greater amounts of sodium and chloride in the urinary foods. In addition, significantly lower urine pH, ammonium, potassium, phosphorus, magnesium, oxalate, citrate, and sulfate were seen with consumption of urinary foods. Given the composition of struvite uroliths, it corresponds that consumption of foods formulated for urinary indications would lead to less magnesium, ammonium, and phosphorus in the urine. Further, these data are consistent with the prior observation that foods with higher levels of magnesium or phosphorus led to an increased risk of struvite urolith formation in cats (23).

No difference in urine calcium concentrations were observed in this study between the foods formulated for urinary and non-urinary indications. Prior observations established that higher dietary sodium results in increased urinary calcium excretion but a lower urinary calcium concentration due to urine dilution (46). This may explain the observation in the present study despite higher sodium in the urinary foods compared with the non-urinary foods and may be an area for future study. Urine dilution may also be contributing to the fewer observed crystals in the urine of cats fed the urinary foods. Since the RSS calculation utilizes fractional excretion of analytes rather than urine specific gravity, urine dilution may be a factor in reduced crystallization, but it is not captured in the RSS calculation.

The initial five-predictor variable model for examining the relationship between RSS and the urine analytes to predict struvite RSS was unsuccessful. However, an eight-predictor variable model selected for predicting the natural log of struvite RSS values was much more successful and indicated that predicted struvite RSS values would increase with increasing urine pH, ammonium, chloride, calcium, phosphorus, and magnesium, and that predicted struvite RSS values would decrease with increasing urine citrate and sulfate. For modeling of CaOx RSS, an eight-predictor variable model accurately predicted CaOx RSS, with predicted CaOx RSS values increasing as urine chloride, calcium, and oxalates increased, and decreasing as urine pH, sodium, phosphorus, citrate, and sulfate increased. Because existing CaOx uroliths cannot be dissolved by a food-based intervention, their prevention is key. Thus, the ability to predict CaOx RSS can help in the determination of which foods would result in less CaOx crystal formation and thus prevent CaOx uroliths. Of note, there is a lack of publications for comparison with this work, pointing to a potential need for further study.

One limitation of using RSS analysis is that there are parameters that are not considered in the RSS algorithm that can have an effect on urolith formation. Dietary protein levels also appear to influence crystallization, as feeding cats a high-protein food (55% crude protein) led to significantly lower urine pH and fewer struvite crystals compared with a lower-protein food (47). Since only one of the foods in the present analysis had a crude protein level > 55%, future work is needed to determine the contribution of high dietary protein to RSS values and crystallization. In addition, supplementation of cat food with long-chain polyunsaturated fatty acids led to a decreased RSS for struvite, fewer struvite crystals, and greater resistance to CaOx crystal formation compared with feeding a food without this supplementation (48). The optimal form of nutrients for urolith prevention may also not be clear, as KHCO_3_ as a potassium source appeared to be more beneficial than KCl for the prevention of CaOx uroliths (49). As we could not determine the form of potassium used in the foods of the present analysis, we cannot evaluate whether this was a contributor to the observations of this study. This analysis was also limited due to its retrospective nature, so specific variables that may be of interest were not varied in the foods tested and so could not be examined as predictors of RSS. It should be noted that statistical significance (or lack of) does not indicate clinical significance, and the differences should be interpreted from a clinical perspective. Another limitation is that these feeding trials were performed on healthy cats, so the results may not be applicable to cats who form uroliths. Nonetheless, RSS as calculated by EQUIL-HL21 was able to show differences in struvite RSS values between consumption of urinary and non-urinary foods, which was supported by the nearly six-fold difference in the observed number of struvite crystals.

In summary, data from this analysis indicate that the RSS values determined via the EQUIL-HL21 program can discriminate between foods formulated for urinary and non-urinary indications. Regression modeling has indicated which urinary analytes contribute to the predicted RSS values for struvite and CaOx.

## Data availability statement

The raw data supporting the conclusions of this article will be made available by the authors, without undue reservation upon reasonable request.

## Ethics statement

The animal studies were approved by Institutional Animal Care and Use Committee, Hill’s Pet Nutrition, Topeka, KS, United States. The studies were conducted in accordance with the local legislation and institutional requirements. Written informed consent was not obtained from the owners for the participation of their animals in this study because animals were owned by Hill’s Pet Nutrition.

## Author contributions

EM and DJ contributed to the conception and design of the study. EM and AM searched the historical trial data. JB performed the statistical analysis. All authors contributed to the development of the manuscript, including interpretation of data, and approved the submitted version.

## Funding

This study received funding from Hill’s Pet Nutrition, Inc.

## Conflict of interest

JB is an employee of Alpha Statistical Consulting, Inc., a subcontractor of Hill’s Pet Nutrition, Inc., and DJ is a former employee of Hill’s Pet Nutrition, Inc.

The Authors declare that this study received funding from Hill’s Pet Nutrition, Inc. The funder had the following involvement in the study: provided funds for the consultants and provided the bio-archive data for analysis.

## Publisher’s note

All claims expressed in this article are solely those of the authors and do not necessarily represent those of their affiliated organizations, or those of the publisher, the editors and the reviewers. Any product that may be evaluated in this article, or claim that may be made by its manufacturer, is not guaranteed or endorsed by the publisher.
