# Supplementary material for: Relative supersaturation values distinguish between feline urinary and non-urinary foods and align with expected urine analytes contributions to uroliths
Source: Front Vet Sci. 2023 Aug 4;10:1167840. doi: 10.3389/fvets.2023.1167840 (PMC10436620; doi:10.3389/fvets.2023.1167840)
Supplement: Supplementary file 3 [file Data_Sheet_3.PDF]

Supplementary Material

Relative supersaturation values distinguish between feline urinary and non-urinary foods and align with expected urine analytes contributions to uroliths

Elizabeth M. Morris*, Allison P. McGrath, John Brejda, Dennis E. Jewell

*** Correspondence:** Elizabeth M. Morris: elizabeth_morris@hillspet.com

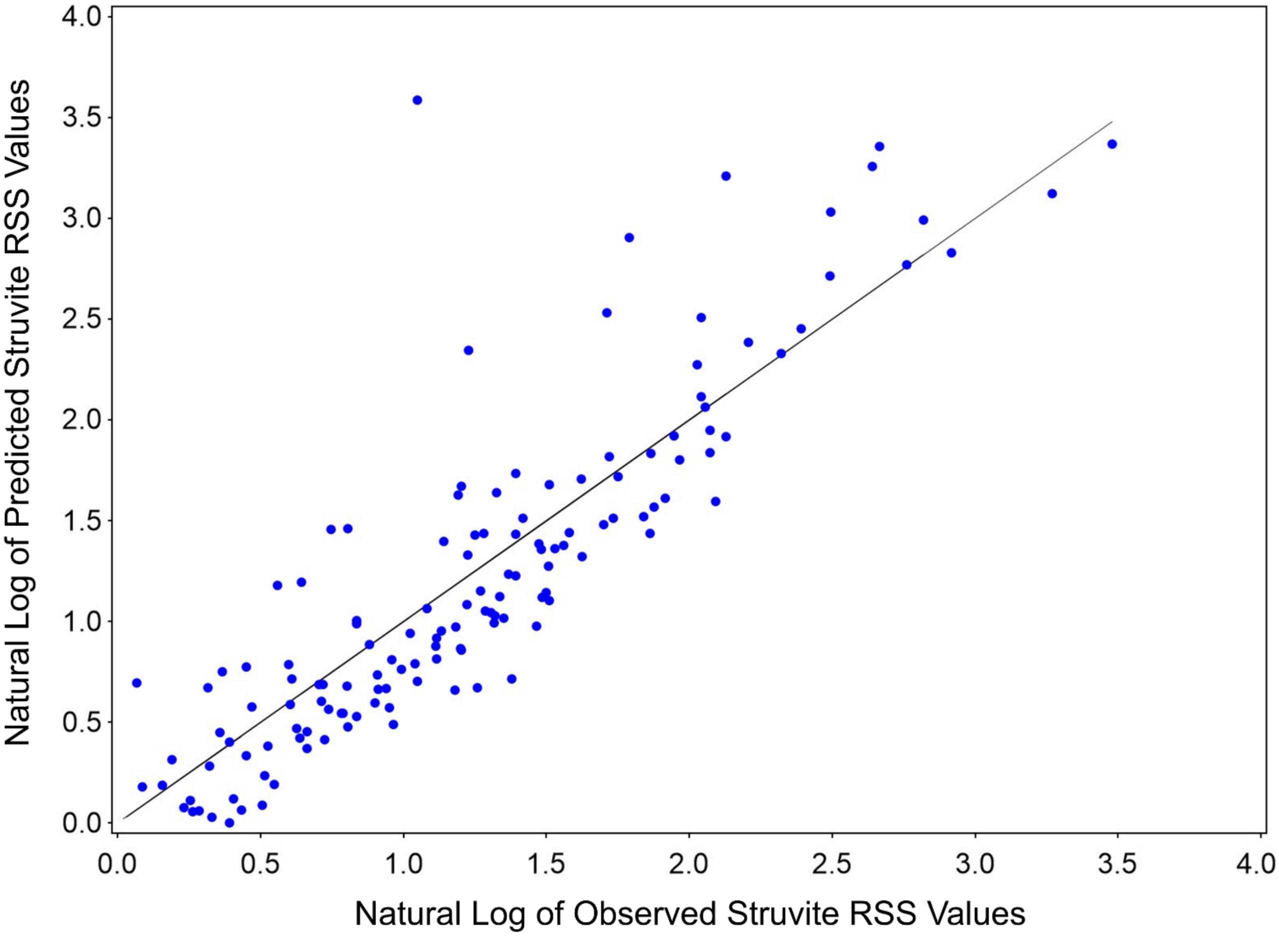

Supplementary Figure 3. Plot of predicted versus observed values for the natural log of struvite relative supersaturation (RSS; blue dots). The solid black line is a 1:1 line for the natural log of observed and predicted struvite RSS values.
