# Supplementary material for: Relative supersaturation values distinguish between feline urinary and non-urinary foods and align with expected urine analytes contributions to uroliths
Source: Front Vet Sci. 2023 Aug 4;10:1167840. doi: 10.3389/fvets.2023.1167840 (PMC10436620; doi:10.3389/fvets.2023.1167840)
Supplement: Supplementary file 6 [file Data_Sheet_6.PDF]

Supplementary Material

Relative supersaturation values distinguish between feline urinary and non-urinary foods and align with expected urine analytes contributions to uroliths

Elizabeth M. Morris*, Allison P. McGrath, John Brejda, Dennis E. Jewell

*** Correspondence:** Elizabeth M. Morris: elizabeth_morris@hillspet.com

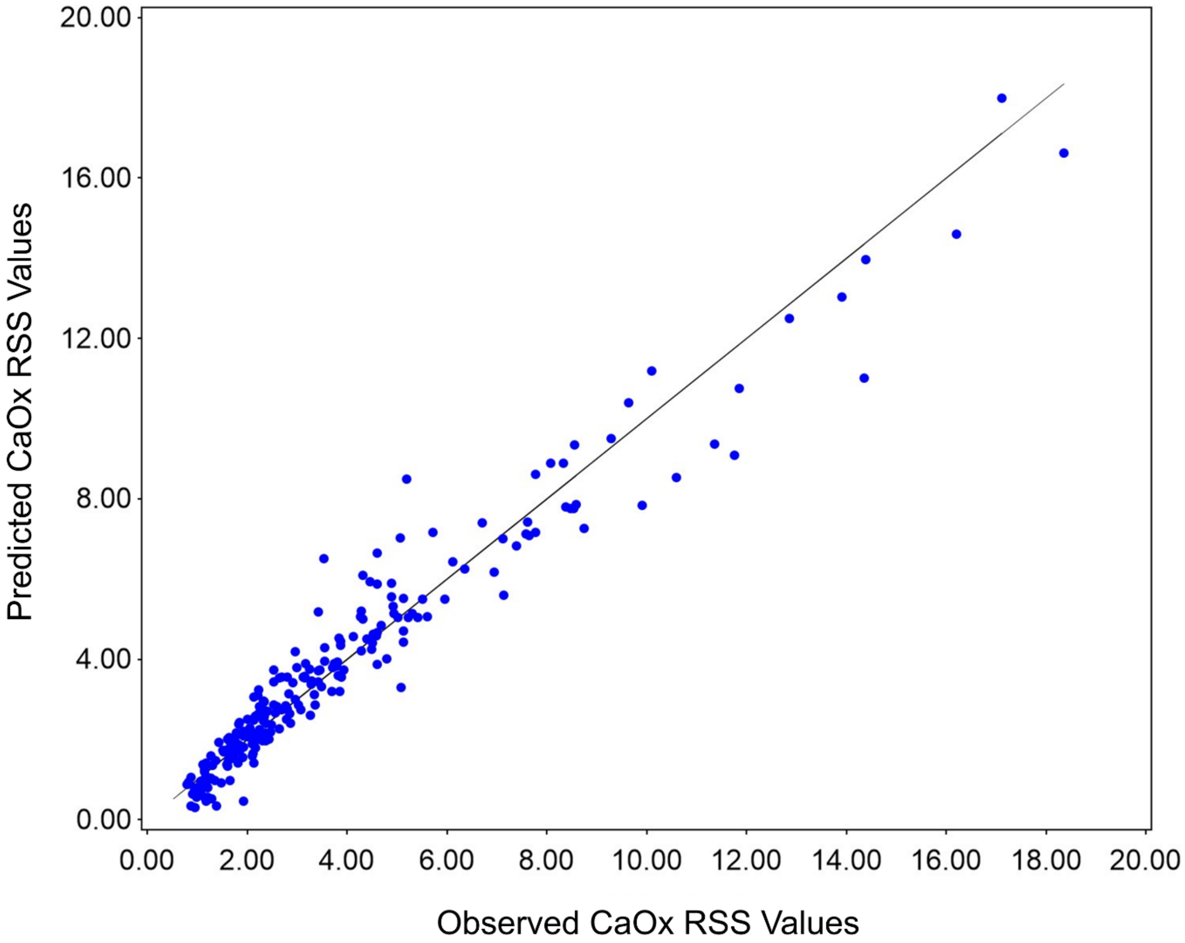

Supplementary Figure 6. Plot of predicted versus observed values from an eight-predictor variable model for predicting calcium oxalate (CaOx) relative supersaturation (RSS) using urine analytes (blue dots). The solid black line is a 1:1 line for observed CaOx RSS values.
