# Supplementary material for: Relative supersaturation values distinguish between feline urinary and non-urinary foods and align with expected urine analytes contributions to uroliths
Source: Front Vet Sci. 2023 Aug 4;10:1167840. doi: 10.3389/fvets.2023.1167840 (PMC10436620; doi:10.3389/fvets.2023.1167840)
Supplement: Supplementary file 8 [file Table_1.pdf]

Supplementary Material

Relative supersaturation values distinguish between feline urinary and non-urinary foods and align with expected urine analytes contributions to uroliths

Elizabeth M. Morris*, Allison P. McGrath, John Brejda, Dennis E. Jewell

*** Correspondence:** Elizabeth M. Morris: elizabeth_morris@hillspet.com

Supplementary Table 1. Nutrient composition of the foods used in the study on a dry matter basis.

Food	Energy, kcal/kg[1]	Crude Protein	Crude Fat	Carbo-hydrate (NFE)	Crude Fiber	Ash	Calcium	Phos-phorus	Potass-ium	Magne-sium	Sodium	Chloride	Sulfate
**Non-Urinary Foods**													
1	4008.6	32.5	13.9	45.2	1.9	6.5	0.99	0.93	0.95	0.15	0.37	0.61	-
2[2]	4117.8	-	-	-	-	-	-	-	-	-	-	-	-
3	5068.0	49.9	37.6	1.0	0.2	11.2	2.22	1.80	0.97	0.11	0.71	1.01	-
4	4077.9	56.9	29.6	0.6	0.4	12.5	2.47	1.97	1.07	0.14	0.88	1.31	-
5	4338.5	32.5	20.2	38.5	3.2	5.7	0.85	0.79	0.75	0.09	0.47	0.85	-
6	4136.3	34.1	21.1	35.1	4.7	5.1	0.80	0.63	0.98	0.10	0.30	0.78	-

Food	Energy, kcal/kg[1]	Crude Protein	Crude Fat	Carbo-hydrate (NFE)	Crude Fiber	Ash	Calcium	Phos-phorus	Potass-ium	Magne-sium	Sodium	Chloride	Sulfate
7	4373.4	31.7	21.6	40.4	1.9	4.4	0.76	0.56	0.79	0.07	0.24	0.41	2.00
8[3]	5225.0	37.2	19.6	34.9	1.4	6.9	1.18	1.02	0.92	0.10	0.40	0.89	0.11
9[2]	3426.2	38.20	9.18	37.7	7.8	7.2	1.12	0.84	0.82	0.08	0.42	0.61	-
10	3972.8	32.3	15.2	43.8	2.0	6.7	1.48	1.23	0.83	0.12	0.36	0.45	-
**Urinary Foods**													
11	5070.6	34.3	14.8	44.9	1.2	4.8	0.71	0.67	0.83	0.07	0.33	0.84	0.31
12	5692.0	42.6	21.3	30.2	0.5	5.3	0.83	0.70	0.86	0.06	0.37	1.02	0.27
13	4480.3	43.5	25.1	23.6	2.5	5.3	0.75	0.80	0.80	0.08	0.34	0.75	-
14a[4]	4169.2	33.0	16.1	45.6	0.5	4.8	0.71	0.67	0.79	0.06	0.33	0.78	-
14b[4]	4380.3	39.4	20.0	24.3	3.0	5.5	0.74	0.72	0.82	0.06	0.31	0.70	-
15	4828.1	34.9	15.5	38.8	3.1	7.7	0.64	0.77	0.98	0.05	1.24	2.40	0.71
16	4960.4	35.0	15.6	39.9	2.6	6.9	0.80	0.71	0.99	0.06	0.85	1.84	0.73
17	4666.9	33.7	15.8	38.3	2.3	8.7	0.93	0.84	1.03	0.08	1.20	2.00	1.81
18	4960.4	35.1	17.1	36.2	2.6	9.0	1.14	0.92	1.01	0.08	1.32	2.11	1.81
19	4784.0	32.6	16.1	40.6	2.0	8.7	0.96	0.89	1.00	0.08	1.37	2.21	1.22

Food	Energy, kcal/kg[1]	Crude Protein	Crude Fat	Carbo-hydrate (NFE)	Crude Fiber	Ash	Calcium	Phos-phorus	Potass-ium	Magne-sium	Sodium	Chloride	Sulfate
20	4136.0	34.2	17.0	38.0	2.5	8.3	0.96	0.88	0.98	0.07	1.31	2.08	-

Units are % unless otherwise indicated.

[1]In the event that energy content was not analyzed in a food, the published metabolizable energy value was used.

[2]Nutritional analysis was not completed for all foods; since these are historical data, analyses could not be completed.

[3]Food 8 was used in two separate feeding trials.

[4]Food 14 was a mix of the dry and wet versions of a product, and the amount of each fed was different for each cat.
